# MASLD treatment—a shift in the paradigm is imminent

**DOI:** 10.3389/fmed.2023.1316284

**Published:** 2023-12-11

**Authors:** Mariana Verdelho Machado

**Affiliations:** ^1^Faculdade de Medicina, Universidade de Lisboa, Lisbon, Portugal; ^2^Hospital de Vila Franca de Xira, Vila Franca de Xira, Portugal

**Keywords:** MASLD, treatment, obesity, weight loss, liver disease-targeted drugs

## Abstract

MASLD prevalence is growing towards the leading cause of end-stage liver disease. Up to today, the most effective treatment is weight loss. Weight loss interventions are moving from lifestyle changes to bariatric surgery or endoscopy, and, more recently, to a new wave of anti-obesity drugs that can compete with bariatric surgery. Liver-targeted therapy is a necessity for those patients who already present liver fibrosis. The field is moving fast, and in the near future, we will testify to a disruptive change in MASLD treatment, similar to the paradigm-shift that occurred for hepatitis C almost one decade ago with direct antiviral agents.

## 1 Introduction

Metabolic-associated steatotic liver disease (MASLD) is a global pandemic, affecting 2 out of 5 persons worldwide (1). Recently, a study on the NHANES database showed a prevalence of steatotic liver disease of 42%, with almost all patients presenting metabolic dysfunction, of whom almost 90% had MASLD and 8% Met-ALD (that is the combination of MASLD with moderate alcohol intake) (2). In the last half-century, its prevalence increased by 50%, with a striking acceleration in its growth in the last two decades (1), when the incidence of MASLD is estimated to have tripled (3). Aligned with these statistics, MASLD is the contributor to the burden of cirrhosis and liver cancer with the most rapid global growth (4), being already the second cause of liver transplantation, and the first among women (5).

The main driver of MASLD is obesity and adiposopathy (6), as well as the metabolic disturbances that come with it such as insulin-resistance/type 2 diabetes mellitus (T2DM), dyslipidemia, and hypertension (7). When considering the treatment of patients with MASLD, we must take into consideration that 90–95% of patients will not progress to liver cirrhosis, and the ones that progress to liver cirrhosis take 20 to 25 years (8). Also, MASLD pathology is a highly dynamic entity, with the possibility to regress, which may occur in the very short time when it comes to steatosis, and longer, but still possible, for fibrosis, when adiposopathy is mitigated. Importantly, patients with MASLD have an almost two-fold increase in mortality compared to the general population, which increases with the prevalence and severity of liver fibrosis, being up to 4-fold in those with F4 fibrosis/cirrhosis (9). The severity of fibrosis associates not only with liver-related mortality but also with overall and cardiovascular mortality (10). The main causes of death are cancer, cardiovascular disease, and only in third place liver disease (9). Remarkably, the obesity-associated risk for cancer seems to be dependent on the development of MASLD (11).

MASLD patients should be managed by multidisciplinary teams composed by hepatologists, endocrinologists, cardiologists, physical and rehabilitation doctors, dietitians, and psychologists (12). The ideal treatment for MASLD should target not only the progression of liver disease but also the metabolic risk factors that promote cardiovascular disease and cancer. Because MASLD is a slowly progressive disease, clinical endpoints are difficult to achieve and assess in clinical trials. As such, the most aspired endpoint is fibrosis reversal, since fibrosis severity is the main prognostic factor in MASLD, not only regarding liver progression and outcomes, as well as extra-hepatic cardiovascular endpoints. Finally, the ideal treatment for MASLD should have a solid safety profile, in order not to induce harm in asymptomatic patients that may continue morbid event-free for decades. Currently, there is still no drug approved for the treatment of MASLD, but we are living in exciting times, with strong joined efforts in the quest to find new efficient drugs.

This review will summarize how MASLD patients should be managed today, as well as the recent advances in the field and what to expect in the near future.

## 2 Management of adiposopathy

MASLD is the hepatic expression of adiposopathy, which occurs when the adipose tissue capacity is surpassed by surplus energy (6). Severe obesity systematically exceeds the adipose tissue capacity (13), but each individual has its own intrinsic threshold, which may be surpassed even in the range of normal body weight index (BMI) (14, 15). Adiposopathy promotes MASLD but also metabolic dysfunction, cardiovascular disease, and cancer. As such, all patients with MASLD, whatever the severity of the pathology, are at risk of increased all-cause mortality, mostly at the expense of cancer and cardiovascular-related mortality (9). Hence, all patients with MASLD should be managed by targeting adiposopathy, decreasing the adipose tissue burden, and improving its function.

Weight loss is an efficient strategy to improve MASLD. Indeed, loss of at least 5% of body weight associates with steatosis regression, 7% loss with metabolic-associated steatohepatitis (MASH) resolution, and 10% or more with fibrosis regression in up to 80% of the patients (16). This could not be equalized, until now, by any drug therapy. Even in patients already with liver cirrhosis, weight loss achieved with a hypocaloric diet and exercise is still beneficial, since it induces a decrease in portal hypertension (17).

### 2.1 Lifestyle interventions

Recent studies with the NHANES database showed that a healthy diet and physical activity synergically protect from MASLD. The protective effect of diet could be virtually explained by lower BMI and waist circumference, whereas the latter would only be responsible for about half the attributable benefits of physical activity (18). Similarly, in patients with MASLD, a healthy diet and physical activity can decrease all-cause and cardiovascular mortality. There is no lower threshold above which physical activity protects from mortality, and any increase in exercise can still have a positive impact on survival (19).

Regarding diet recommendations, patients should be advised to engage in a hypocaloric diet, with a 500 to 1,000 kcal deficit, in order to promote weight loss. The relative importance of energy intake quantity over the quality of nutrients in the diet is debatable, with epidemiological studies showing discrepant results (20). However, there is strong evidence that high fructose consumption promotes MASLD development and progression (21). Furthermore, the type of fat consumed may have a role, with some evidence that high cholesterol and saturated fatty acids intake promotes MASLD, fibrosis, and hepatocellular carcinoma, whereas omega-3 polyunsaturated fatty acids showed a potential role in hepatocellular carcinoma protection (22–24). Finally, consumption of animal proteins, particularly from red meat seems to be associated with insulin resistance and MASLD (25).

Different diets have been proposed, the most accepted one being the Mediterranean diet (MD). The MD is a health-promoting diet that consists of a high consumption of plant-based foods such as vegetables and fruits, whole grains, seeds, nuts, and legumes; and a low consumption of sugars and refined carbohydrates. It favors fish over meat, being particularly scarce in red meat. The primary source of fat comes from monounsaturated fatty acids-rich olive oil (26). Small studies and meta-analyses suggest that the MD is associated with an improvement in liver enzymes, steatosis, and even liver fibrosis evaluated by non-invasive tests (NIT) (27, 28). Importantly, in the general population and in MASLD patients, MD seems to be associated with a lower risk of T2DM, cardiovascular and cancer mortality, including from liver cancer (29–31).

Another popular diet is intermittent fasting. These diets allow *ad libitum* energy intake but are restricted to a limited time window. Focusing on an eating window rather than caloric intake, has the advantage of potentially higher adherence compared to calorie-restricted diets, since simply skipping a meal can promote by itself a restriction in caloric intake and weight loss (32). Intermittent fasting also seems to improve metabolic dysfunction with better glucose and blood pressure control (33), liver steatosis, and liver fibrosis assessed by NIT (34). However, the level of evidence resumes small studies with weak endpoints, and it is not more effective than other diets with calorie restriction regarding weight loss or metabolic dysfunction (35). Also, when engaging in intermittent fasting, one should take into consideration that the choice of meal to skip may matter, since diurnal circadian rhythms may have an impact on health. For example, epidemiological studies suggest that skipping breakfast was associated with overweight and obesity (36), a higher risk of T2DM (37), cardiovascular mortality (38), as well as, gastrointestinal and liver cancer (39).

Coffee consumption provided that sugar/sweeteners are not added, seems to have a protective role, with a reported association between intake of at least two coffees a day with a lower risk of steatosis and liver fibrosis (40, 41), and 3 coffees with a decreased risk for hepatocellular carcinoma (42). The beneficial effects are transversal for regular and decaffeinated coffee (43).

Regarding alcohol intake, even though there is no robust scientific evidence to recommend complete abstinence in all MASLD patients, we should not advise patients to drink alcohol. Large epidemiological studies even suggest that in patients with MASLD, very mild alcohol consumption (that is, less than one drink a day) was associated with a decreased all-cause mortality, but only in nonsmokers and without significant fibrosis (44, 45). Having said that, alcohol and metabolic dysfunction are synergic in inducing liver disease (46–48), and a recent systematic review suggested that any alcohol intake might increase the risk of progression of liver disease in MASLD patients (49). In patients with cirrhosis, any alcohol intake was associated with increased mortality (50) and risk of hepatocellular carcinoma (51).

Physical activity, particularly recreational and not occupational, associated with weight loss and a protective effect against liver steatosis (52), even when weight loss was not achieved. Both aerobic and anaerobic exercises should be advised, and any increase in physical activity seems beneficial, even though the goal should be at least 45 min of moderate-intensity exercise, 3 times per week (53).

Interventions in lifestyle are hindered by a low success rate in achieving weight loss, lower than 10% (16). Furthermore, only up to one-fourth of those achieving weight loss are able to maintain the weight, and 60% of patients regain weight within the first year (54).

These statistics should not persuade us to enroll patients in lifestyle interventions. Indeed, even when patients regain weight after achieving weight loss during lifestyle interventions, the beneficial effects of the transient weight loss on liver steatosis and metabolic dysfunction seems to persist for at least 2 years (55).

### 2.2 Bariatric surgery

Bariatric or metabolic surgery is an approved intervention to manage patients with morbid obesity (BMI at least 40 kg/m^2^), grade II obesity (BMI 34.9 to 40 kg/m^2^) with at least one weight-related comorbidity, or patients with T2DM and grade I obesity who maintain poor metabolic control after lifestyle intervention and pharmacotherapy (56). Indeed, compared with conventional treatment, bariatric surgery associated with long-term weight loss, decreased incidence of T2DM, improvement in glucose metabolism control in diabetics translating in lower rates of diabetes-related complications (57), and an up to 50% decrease in all-cause and cardiovascular mortality (58, 59) with an increase in life expectancy of 6 years (up to 9 years in diabetic patients) (60).

Bariatric surgery is also effective in the treatment of MASLD, inducing long-term effects that sustain at least 5 years. Observational studies suggested that bariatric surgery promotes steatosis resolution, MASH resolution without worsening fibrosis in around 80%, and fibrosis regression in 70%. More than 50% of the patients may achieve complete fibrosis resolution, even the ones with advanced fibrosis at baseline. The beneficial effects seem dependent on weight loss (61–63). A recent open-label trial randomized almost 300 patients with MASH for lifestyle intervention or bariatric surgery, and showed that surgery was associated with a one-year 70% higher chance of achieving fibrosis improvement of at least one stage and with a 50% decreased risk of worsening fibrosis (64). Importantly, in MASLD patients, bariatric surgery was associated with a decreased risk of major liver and cardiovascular outcomes (65, 66).

There are two types of bariatric surgery: (1) Restrictive, which restricts the calorie intake through decreasing the stomach capacity; and (2) Restrictive and Malabsorptive, which bypass the proximal small bowel leading to a decreased absorptive surface and a hormonal effect that improves insulin resistance. Examples of restrictive surgeries are Adjustable Gastric Banding and Sleeve Gastrectomy. Examples of restrictive and malabsorptive surgeries are Roux-en-Y Gastric Bypass and Biliopancreatic Diversion with Duodenal Switch (67). Restrictive surgeries induce less weight loss but at the expense of lower adverse effects as compared with malabsorptive surgeries (67). The effect on MASLD seems dependent on the type of surgery performed. Indeed, adjustable gastric banding seems less effective in improving liver histology (68), whereas sleeve gastrectomy and gastric bypass seem similarly effective (69–71).

The complication rate of bariatric surgery does not seem to increase in patients with MASH (72). However, bariatric surgery should be proposed with caution to patients with liver cirrhosis, since mortality rates surpass 1%. Decompensated cirrhosis is an absolute contraindication for bariatric surgery since mortality rates increase to almost 20% (73). In the context of liver transplantation, surgery could be offered before the transplant in well-compensated cirrhotics. Furthermore, the Mayo Clinic is undergoing a prospective program of simultaneous liver transplantation and sleeve gastrectomy with promising results (74, 75). Bariatric surgery after liver transplantation also seems feasible, even though the level of evidence is low (76).

### 2.3 Endoscopic bariatric interventions

The field of endoscopic bariatric interventions has evolved dramatically in the past years, with the development of several new interventions. We have now available intragastric devices that restrict food intake (balloons, endoscopic sleeve gastroplasty, and aspiration therapy) and small bowel devices that target metabolic profile and insulin resistance (endobarrier and duodenal mucosal resurfacing). Intragastric balloons are the oldest technology and work by filling the stomach with a balloon, which decreases its capacity promoting early satiety. Endoscopic sleeve gastroplasty consists of a decrease in stomach size by applying full-thickness bites with a suturing device causing apposition of tissue along the greater curvature. Aspiration therapy consists of applying a percutaneous gastrostomy tube, which allows the partial drainage of the gastric food content after a meal. Endobarrier consists of the application of a plastic liner, 60 cm long, anchored to the duodenal bulb, which prevents duodenal absorption. Last, duodenal mucosa resurfacing consists of the hydrothermal ablation of 10 cm of the duodenal mucosa, which decreases its absorptive function (67).

Even though the level of evidence for the beneficial effects of these interventions in the management of MASLD patients is low, a recent meta-analysis with 863 patients, suggests that globally these procedures may result in histologic improvement (77). The intragastric balloon seems to have only a transient effect on weight, with most patients regaining weight after balloon removal (78), and as such it does not seem an adequate long-term therapy (79). On the contrary, a study with a 2-year follow-up of obese MASLD patients submitted to endoscopic sleeve gastroplasty did show a sustained effect on weight, as well as an improvement in liver histology, with 20% of the patients that presented baseline F3-F4 fibrosis regressing for F0-F2 fibrosis (80). After these encouraging results, a randomized controlled trial comparing surgical versus endoscopic gastric sleeve interventions, the TESLA-NASH study, is ongoing (81). Regarding endobarrier and duodenal mucosa resurfacing, both have shown, in preliminary studies, to improve steatosis and fibrosis assessed by NIT (82, 83).

### 2.4 Weight loss drugs

The pharmacological treatment of obesity is undergoing exciting advances in the last 5 years. The oldest generation of anti-obesity drugs could achieve a very mild weight loss of 3 to 8% (84). The first change in paradigm was the realization that anti-diabetic drugs that act as glucacon-like peptide-1 (GLP-1) receptor agonists were able to promote weight loss through its pleiotropic action promoting satiety, delaying gastric emptying, aside from its metabolic effects (85). One example is liraglutide, which showed in the SCALE studies, that it could promote weight loss of 6–8% of body weight, at a dose of 3.0 mg daily (86, 87). Liraglutide also showed in a small phase 2 study, the LEAN study, with 52 patients, to induce higher resolution of steatohepatitis (39% vs. 9%) and lower progression of fibrosis (9% vs. 36%) compared to placebo, even though it did not associate with improvement in fibrosis (88). Histological response was not associated with higher weight loss, suggesting that the beneficial effects in the liver surpass its effects promoting weight loss.

Semaglutide, another GLP-1 receptor agonist, is more potent than liraglutide in promoting weight loss, at the dose of 2.4 mg per week (89, 90). The STEP studies showed an average weight loss of 12% (91–95). Besides its effects on glucose control, it also improved lipid profile and blood pressure, even when compared to patients losing the same weight on placebo (91, 96). These metabolic effects may explain why semaglutide treatment is associated with an almost 25% decrease in major adverse cardiovascular events, decreased all-cause and cardiovascular mortality (97–99). Semaglutide was already studied in phase 2 and 3 studies as a treatment for MASLD. A phase 2b study enrolled 320 patients with MASH and fibrosis F1 to F3, who were treated with increasing doses of semaglutide or placebo (100). The highest dose of semaglutide was 0.4 mg per week, which is much lower than the dose used to treat obesity. It reached its primary endpoint with two times the proportion of MASH resolution compared to placebo (40% vs. 17%). It did not promote fibrosis improvement, even though there was a dose-dependent lower probability of worsening fibrosis. Subsequently, other phase 2 study, enrolling 71 patients with MASLD-associated cirrhosis, treated with 2.4 mg per week of semaglutide, controlled with placebo, failed to show an improvement in fibrosis or MASH (101). There was a skew for worse fibrosis (higher percentage of patients with ISHAK grade 6 and higher hepatic collagen proportion), but we might assume that after cirrhosis development semaglutide is no longer effective. A phase 3 trial is ongoing, the ESSENCE trial (NCT04822181), that started, in 2021, recruiting of patients with MASH and stage F2 or F3 fibrosis, treated with semaglutide 2.4 mg per week, controlled with placebo. Histological endpoints will be evaluated at weeks 72 and 240, as well as clinical endpoints such as histological progression into cirrhosis, hepatic decompensation, death or liver transplantation.

The subsequent advance in anti-obesity treatment was the development of dual incretins, such as tirzepatide, a dual GLP-1 and glucose-dependent insulinotropic polypeptide (GIP) agonist, and cotadutide, a dual GLP-1 and glucagon agonist. Tirzepatide adds the beneficial effects of GLP-1 to the enhancement in adipose tissue function mediated by GIP. A set of studies, the SURPASS-1/5, designed to evaluate tirzepatide as a treatment for T2DM, found it could induce impressive weight loss, of around 20% of body weight (102–106). These results were replicated in a phase 3 clinical trial designed to evaluate weight loss in patients without T2DM, the SURMOUNT-1 (84). A substudy of SURPASS-3, a randomized clinical trial in T2DM patients on metformin, which enrolled 296 patients, showed that tirzepatide treatment associated with an almost 50% relative decrease in liver fat content (LFC) assessed by MRI-PDFF that correlated with weight loss and decreases in visceral and abdominal subcutaneous tissue (107). Furthermore, a post-hoc analysis of a phase 2 trial in T2DM patients, also reported an association between treatment with tirzepatide for 26 weeks and a decrease in NIT of hepatocyte apoptosis (K18 fragments) and fibrosis (propeptide of type III collagen, ProC3) (108). It is currently ongoing a phase 2 study specifically in MASH patients, with histological endpoints, the SYNERGY NASH study (NCT0416673).

Several dual GLP-1 and glucagon agonists are in evaluation with potential benefits in MASH: cotadutide, efinopegdutide, and pemvidutide. The glucagon activity adds potential value since it promotes hepatic lipolysis and fat mobilization, as well as increases energy expenditure. However, it has the potential to increase glucose and insulin levels (109). Cotadutide is a peptide with the glucagon sequence modified at some amino-acids and with the addition of a palmitic fatty acid side chain that prolongs its activity. Those modifications resulted in a dual GLP-1 receptor and glucagon receptor agonist activity, which is 2-fold more potent for GLP-1 receptor (110). Cotadutide showed to promote similar weight loss as liraglutide, and lower than semaglutide, but it seems better than liraglutide in promoting a decrease of liver fibrosis when assessed by non-invasive scores and ProC3, in overweight/obese T2DM patients (111, 112). Cotadutide treatment also resulted in an improvement in lipid profile, decreasing LDL-cholesterol and triglycerides (111). Efinopegdutide is a synthetic peptide of oxyntomodulin (a peptide product of the proglucagon gene produced in the small bowel in response to food ingestion) conjugated to the constant region of human IgG4, which results in a longer half-life. It has dual GLP-1 and glucagon agonistic properties, with a relative potency of 2:1 (113). It achieved a weight loss of around 10% in 6 months at a dose of 10 mg per week, performing better than liraglutide and semaglutide regarding weight loss and improvement in lipid profile (109, 113, 114). However, efinopegdutide was less tolerated than liraglutide, and it did not improve glycated hemoglobin, increasing glucose and insulin levels (109, 114). A phase 2a study in 145 patients with MASLD showed efinopegdutide treatment to induce an astonishing relative decrease of LFC by MRI-PDFF, on average higher than 70%, which was superior to the one achieved by semaglutide even at the same weight loss (113). Pemvidutide is a peptide with a balanced GLP-1 and glucagon agonist activity, conjugated with a glycolipid surfactant that prolongs its half-life, allowing weekly administrations (115). After enthusiastic results in preclinical mouse models of MASH, as compared with semaglutide and elafibranor (115), pemvidutide was recently evaluated in a phase 1b study in patients with NAFLD, showing again impressive improvement in LFC of around 75% at 6 months, as well as of liver volume (116). A phase 2b study, the IMPACT NASH trial (NCT05989711), is ongoing, in 190 NASH patients, with histological endpoints, which is expected to be presented in 2025.

More recently, a triple hormone agonist approach allowed a step forward in weight loss therapy. Retatrutide is a triple GLP-1, GIP, and glucagon agonist, which showed a dose-dependent weight loss (117), achieving weight loss of around 25% of body weight at a 12 mg dose per week during 48 weeks (118). A subgroup analysis in patients with MASLD showed an average of over 80% decrease of liver fat content, with all patients decreasing at least 30% and around 90% achieving MASLD resolution, at 8 mg or 12 mg dose (119).

In conclusion, there has been a paradigm shift in the pharmacological treatment of obesity, in the post-incretin era, which might dethrone bariatric surgery in the near future.

## 3 Drugs approved for other indications that may have an impact in MASLD

Current guidelines consider two drugs on the market that are approved for other conditions: vitamin E and pioglitazone, under specific circumstances (12).

Vitamin E was studied for MASH in the PIVENS trial. The PIVENS trial randomized 247 non-diabetic patients with non-cirrhotic MASH to either vitamin E 800 IU per day, pioglitazone 30 mg per day, or placebo, during 96 weeks (120). The results fell short since there was no improvement in liver fibrosis. However, there was an improvement in steatohepatitis and hepatocyte stress/ballooning, independent of weight loss. Those results placed vitamin E as an attractive drug, in an era when there are no approved drugs for MASLD, in patients with steatohepatitis who could not achieve weight loss. However, vitamin E treatment for MASH was never consensual, since the benefit was weak and there are some concerns regarding safety such as a possible increase in mortality for doses higher than 400 IU per day, which was not confirmed in prospective studies (121). There remains conflicting evidence for prostate cancer (122) and for a possible increased risk for hemorrhagic stroke (123). Indeed, there is limited evidence from some observational studies and 2 out of 4 randomized controlled trials suggesting a mild increase in the risk for hemorrhagic stroke and a possible decreased risk in ischemic stroke (121), which was attributed to its antiplatelet actions (124). Subsequently, 3 meta-analyses of smaller studies on vitamin E, suggest not only a benefit in steatosis and inflammation, as well as in liver fibrosis (125–127), particularly for doses higher than 500 IU per day, for longer than 20 months (127). Lastly, a retrospective study that evaluated 180 patients with MASH and advanced fibrosis (F3 or F4) treated with vitamin E 800 IU/day for longer than 2 years, as compared with 90 propensity-matched controls, showed vitamin E to be associated with an increased transplant-free survival (90% vs. 78% at 10 years, with a number needed to treat, NNT, of just 4.28) and decreased rates of hepatic decompensation (37% vs. 62% at 10 years, with a NNT 6.43) (128).

Pioglitazone was also studied in the PIVENS study, at a dose of 30 mg/day, and, similarly to vitamin E, failed to demonstrate improvement in liver fibrosis, even though it did improve steatohepatitis (120). Subsequently, smaller studies and a meta-analysis did show an improvement in liver fibrosis, but only in patients with T2DM (129–131). There are also some safety concerns, such as weight gain, water retention, increased risk for osteoporosis (132), and bladder cancer (133). Of note, pioglitazone does not induce heart failure, on the contrary, it even seems to improve cardiac function and reduce cardiovascular major events (134–136). However, it does increase the rate of hospitalization in patients with heart failure by promoting water retention (137). Finally, regarding the risk of bladder cancer, the NNT to potentially cause an additional case of bladder cancer is 899 to 6,380 (138), which is in convincing contrast to the NNT of 2–12 to reverse a case of MASH (139). PXL065 is a promising deuterium-stabilized R-pioglitazone stereoisomer that retains the effects of pioglitazone in glucose metabolism and in the liver, but has minimal PPAR-γ activity and hence does not promote weight gain or water retention, unlike the S-stereoisomer of pioglitazone (140). Recently, a phase 2 placebo-controlled study, the DESTINY-1, evaluated increasing doses of PXL065 in 117 patients with fibrotic (F1 to F3) MASH. It showed promising results with a dose–response increased probability of improvement in at least one stage in fibrosis, which was associated with an improvement in glucose control and adipocyte function (expressed as an increase in adiponectin), without an increase in body weight or peripheral edema (141).

Another class of anti-diabetics, sodium-glucose cotransporter-2 inhibitors (SGLT-2i) may also be of benefit, even though studies with strong endpoints including liver biopsy are lacking. Treatment with SGLT-2i drugs, such as empagliflozin, seems to induce mild weight loss, as well as important reductions in liver fat content (over 20% relative decrease) and might decrease the risk of fibrosis progression according to small studies with NIT’s assessment of liver fibrosis (142, 143). Importantly, SGLT-2i have important cardiorenal protective effects, being associated with overall and cardiovascular mortality (144–147).

## 4 New drugs in development

Intense research is ongoing in the quest to find drugs that reach the requisites for FDA approval for MASLD. The landscape of clinical trials is wide, and drugs must demonstrate growing robustness when moving toward phase 3 studies. After safety phase 1 studies, phase 2a studies rely on non-invasive endpoints after 24 weeks, phase 2b on histological endpoints in 6 to 24 months, and phase 3 on clinical outcomes after up to 5 years. For FDA approval, a drug must demonstrate hard clinical endpoints such as progression into cirrhosis and hepatic decompensation (148). However, FDA might give conditional approval if, in phase 2 studies, the drug is able to resolve MASH without fibrosis worsening or improve fibrosis without MASH worsening (149). Up to today, no drug has been approved by the FDA.

Several new drugs have failed the requirements when already in phase 2 or 3 studies, which acted in different mechanisms: the metabolism-modulator elafibranor (PPAR-α/δ agonist) (150, 151), the anti-inflammatory cenicriviroc (CCR-2/5 agonist) (152, 153), the apoptosis inhibitor selonsertib (ASK-1 inhibitor) (154), and the anti-fibrotic simtuzumab (antibody against LOXL-2) (155). The most recent drug to be discontinued, even after a positive phase 3 trial, is obeticholic acid (OCA).

Five drugs have now reached phase III trials: OCA, semaglutide, lanifibranor (a pan-PPAR agonist), resmetirom (a thyroid hormone receptor-β, THR-β, agonist), and efruxifermin (a FGF-21 agonist) (Tables 1, 2).

**Table 1:** Glossary of clinical trials in MASLD/MASH.

Trial	Type of trial	Patients	Intervention	Duration	Main findings
ALPINE 2/3 (156)	2b	171 patients with fibrotic (F1-F3) MASH	Adalfermin 0.3 mg, 1.0 mg or 3 mg/day vs. placebo	24 weeks	Did not meet primary endpoints of histological improvement of fibrosis without worsening MASH Dose–response for resolution of MASH (11, 18, and 22% vs. 6% for placebo), relative LFC (25, 38, and 59% vs. 15% for placebo), aminotransferases and ProC3
ARMOR	3	150 patients with fibrotic (F1-F3) MASH	Aramchol 300 mg bid vs. placebo	52 weeks	Ongoing
ARREST (157)	2b	247 patients with fibrotic (F1-F3) MASH	Aramchol 400 mg or 600 mg/day vs. placebo	52 weeks	Did not meet primary endpoint of decrease in relative LFC Did not achieve histological improvement of fibrosis without worsening MASH or MASH resolution without fibrosis worsening
BALANCED (158)	2a	80 patients with fibrotic (F1-F3) MASH	Efruxifermin 28 mg, 50 mg or 70 mg/week vs. placebo	16 weeks	Dose–response decrease in relative LFC: 63, 71 and 72% vs. 0.3% for placebo Decrease in liver enzymes and fibrosis by NIT’s (ELF and ProC3)
CENTAUR (139)	2b	171 patients with fibrotic (F1-F3) MASH	Cenicriviroc 150 mg/day vs. placebo	24 months	Non-significant higher proportion of patients achieved histological improvement of fibrosis without worsening MASH (2-% vs. 11%)
CONTROL (159)	2	84 patients with MASH	OCA 5 mg, 10 mg or 25 mg/day + atorvastatin 10 mg/day after week 4	16 weeks	OCA-induced increases in LDL-cholesterol in patients with MASH were mitigated with atovastatin
DESTINY-1 (133)	2	117 patients with fibrotic (F1-F3) MASH	PXL065 7.5 mg, 15 mg, 22.5 mg/day vs. placebo	36 weeks	Decrease in LFC for all doses (23, 19, 21% vs. 2% increase for placebo) Trend to improvement of fibrosis by NIT’s (PIIINP and NAFLD Fibrosis Score) Improvement in HbA1c and insulin sensitivity. No effect on body weight
ENLIVEN (160)	2b	219 patients with fibrotic (F2-F3) MASH	Pegbelfermin 15 mg or 30 mg/week or 44 mg/q2week vs. placebo	24 weeks	Higher proportion of fibrosis improvement without worsening of MASH (22, 26, and 27% vs. 7% for placebo), and of MASH resolution without worsening fibrosis (37, 23, and 26% vs. 2% for placebo) Improvement of LFC and fibrosis by NIT’s (liver stiffness and ProC3)
ESSENCE	3	1,200 patients with fibrotic (F2-F3) MASH	Semaglutide 2.4 mg/week vs. placebo	72 and 240 weeks	Ongoing
FALCON-1 (161)	2b	197 patients with MASH and bridging fibrosis	Pegbelfermin 10 mg, 20 mg or 40 mg/week vs. placebo	48 weeks	Did not meet primary endpoints of histological improvement of fibrosis without worsening MASH or MASH resolution without worsening fibrosis Dose–response higher proportion of patients with decrease ≥30% MRI-PDFF and ≥ 15% MRE
FALCON-2 (162)	2b	154 patients with MASH and compensated cirrhosis	Pegbelfermin 10 mg, 20 mg or 40 mg/week vs. placebo	48 weeks	Did not meet primary endpoints of histological improvement of fibrosis without worsening MASH or decrease in collagen proportionate area No difference in the proportion of patients achieving decrease ≥30% MRI-PDFF and ≥ 15% MRE
FASCINATE-1 (163)	2a	99 patients with biopsy-confirmed MASH or LFC ≥ 8% + MRE ≥ 2.5	Denifanstat 25 mg or 50 mg/day vs. placebo	12 weeks	Dose–response decrease in relative LFC (10 and 28% vs. 4.5% increase for placebo) Decrease in fibrosis by NITs (ProC3 and TIMP-1) only with the highest dose
FASCINATE-2 (164)	2b	168 patients with fibrotic (F1-F3) MASH	Denifanstat 50 mg/day vs. placebo	26 weeks	Decrease in relative LFC compared with placebo (34% vs. 1.5%) Decrease in fibrosis by NITs (ProC3 and ELF score)
FLIGHT-FXR (165, 166)	2	152 patients with MASH	Tropifexor 140ug or 200ug/day vs. placebo	48 weeks	Did not achieve histological improvement in MASH or fibrosis according to NASH-CRN scoring, but did achieve overall and perisinusoidal liver fibrosis in patients F2/F3 baseline, when assessed by digital pathology (SHG/TPEF) with artificial intelligence
FLINT (154)	2	283 patients with fibrotic (F1-F3) MASH	OCA 25 mg/day vs. placebo	72 weeks	Higher proportion of fibrosis improvement without worsening of MASH (35% vs. 19% for placebo), and of MASH resolution without worsening fibrosis (22% vs. 13% for placebo) Pruritus in almost one-fourth of the patients, worsened insulin resistance and lipid profile
GOLDEN 505 (136)	2b	276 patients with fibrotic (F1-F3) MASH	Elafibranor 80 mg or 120 mg/day vs. placebo	52 weeks	Did not meet primary endpoints of NASH resolution without worsening fibrosis, except in a post-hoc analysis for patients with NAS ≥ 4 (13–19% vs. 9% for placebo) Fibrosis improvement in elafibranor responders Improved cardiometabolic risk profile
HARMONY (167)	2b	Non-cirrhotic fibrotic (F1-F3) MASH	Efruxifermin 28 mg or 50 mg/week vs. placebo	24 weeks	Dose–response higher proportion of fibrosis improvement without worsening of MASH (39 and 41% vs. 20% for placebo), and of MASH resolution without worsening fibrosis (47 and 76% vs. 15% for placebo) Improvement of LFC, weight, insulin sensitivity and lipid profile
IMPACT NASH	2b	190 patients with fibrotic (F2-F3) MASH	Pemvidutide 1.2 mg or 1.8 mg/week vs. placebo	24 and 48 weeks	Ongoing
LEAN (82)	2	52 overweight patients with MASH	Liraglutide 1.8 mg/day vs. placebo	48 weeks	Compared with placebo, liraglutide associated with higher proportion of patients with MASH resolution (39% vs. 9%) and lower with fibrosis progression (9% vs. 36%)
MAESTRO- NASH (168)	3	966 patients with fibrotic (F1-F3) MASH	Resmetirom 80 mg or 100 mg/day vs. placebo	52 weeks	Dose–response higher proportion of fibrosis improvement without worsening of MASH (24 and 26% vs. 14% for placebo), and of MASH resolution without worsening fibrosis (26 and 30% vs. 10% for placebo) Improvement of LFC, fibrosis by NIT’s, liver and spleen volume
NATIVE (169)	2b	247 patients with fibrotic (F1-F3) MASH	Lanifibranor 800 mg or 1,200 mg/day vs. placebo	24 weeks	Dose–response higher proportion of fibrosis improvement without worsening of MASH (39 and 48% vs. 29% for placebo), and of MASH resolution without worsening fibrosis (25 and 36% vs. 7% for placebo) Increase in adiponectin despite weight gain
NATIV3	3	MASH + significant fibrosis (F2-F3)	Lanifibranor 800 mg or 1,200 mg/day vs. placebo	72 weeks	Ongoing
NAVIGATE	2/3	MASH-cirrhosis	Belapectin 2 mg or 4 mg/qow vs. placebo	18 months	Primary endpoint is prevention of esophageal varices Ongoing
PIVENS (113)	3	247 patients with fibrotic (F1-F3) MASH	Pioglitazone 30 mg/day or Vitamin E 800 IU/day vs. placebo	96 weeks	Higher proportion of improvement in MASH in Vitamin E (43%) and Pioglitazone (35%) compared with placebo (9%) Both Vitamin E and Pioglitazone failed to promote fibrosis improvement
REGENERATE (170, 171)	3	2,477 fibrotic (F1-F3) MASH	OCA 10 mg or 25 mg/day vs. placebo	18 months	In the subgroup of 931 patients with fibrosis F2-F3, higher proportion of fibrosis improvement without worsening of MASH (14 and 22% vs. 10% for placebo), and of MASH resolution without worsening fibrosis (6% vs. 3.5% for placebo) Pruritus in almost one-third of patients for 10 mg and 50% for 25 mg arms
RESOLVE-IT (137)	3	1,070 patients with fibrotic (F1-F3) MASH	Elafibranor 120 mg/day vs. placebo	18 months	Did not meet primary endpoints of histological improvement of fibrosis without worsening MASH or MASH resolution without worsening fibrosis Clinical outcomes yet to be known
REVERSE	3	919 patients with compensated MASH-associated cirrhosis	OCA 10 mg or 25 mg/day vs. placebo	72 weeks	Did not meet primary endpoints of histological improvement of fibrosis without worsening MASH Pruritus up to 57% and increased risk of gallstones
STELLAR-3/4 (140)	3	802 patients with F3 and 877 with F4 fibrosis and MASH	Selonsertib 6 mg or 18 mg/day vs. placebo	48 weeks	Did not meet primary endpoints of histological improvement of fibrosis without worsening MASH or MASH resolution without worsening fibrosis
SYNCHRONY	3	Patients with MASH	Efruxifermin		3 clinical trials ongoing: histology, real-world, and outcomes
SYNERGY NASH study	2b	196 patients with fibrotic (F2-F3) MASH	Tirzepatide 10 mg or 15 mg/week vs. placebo	52 weeks	Ongoing
TESLA-NASH	2	30 obese ± MS with non-cirrhotic NASH	Endoscopic sleeve gastroplasty vs. laparoscopic sleeve gastrectomy	96 weeks	
VOYAGE	2b	167 patients with fibrotic (F1-F3) MASH	VK2809 1 mg or 2.5 mg/day, 5 mg or 10mgqod vs. placebo	52 weeks	All doses resulted in a decrease in relative LFC (13, 41, 33 and 48%) at 12 weeks Still ongoing evaluation of histological response at week 52

LFC, liver fat content by MRI-PDFF; MRE, magnetic resonance elastography; NASH-CRN, nonalcoholic steatohepatitis clinical research network; OCA, obethicholic acid; PXL065, deuterium-stabilized R-pioglitaxone; SHG, second harmonic generation; TPEF, two-photon excitation fluorescence.

**Table 2:** Summary of the main results of drugs in phase 3 development for MASH treatment.

	Obeticholic acid	Semaglutide	Lanifibranor	Resmetiron	Efruxifermin
Phase III studies status	Interim analysis	Ongoing	Ongoing	Interim analysis	Ongoing
Patients	MASLD F2-F3 or F4	MASLD F1-F3 or F4	MASLD F1-F3	MASLD F1-F3	MASLD
Effective dose	25 mg/day	0.4 mg/week if F1-F3 2.4 mg/week if F4	1,200 mg/day	80 mg or 100 mg/day	28 mg or 50 mg/week
Treatment duration	72 weeks	72 weeks if F1-F3 48 weeks if F4	24 weeks	52 weeks	24 weeks
Steatosis improvement		Placebo: 26% of patients 0.4 mg: 63% of patients	Placebo: 26% of patients 120 mg: 65% of patients	Placebo: 14% relative ↓ 80 mg: 50% relative ↓	Placebo: 0.3% relative ↓ 28 mg: 65% relative ↓ 50 mg: 71% relative ↓ (at 12 weeks)
MASH resolution without fibrosis worsening	Placebo: 3.5% of patients 25 mg: 6.5% of patients	Placebo: 17% of patients 0.4 mg: 59% of patients No effect if F4	Placebo: 22% of patients 120 mg: 49% of patients	Placebo: 10% of patients 80 mg: 26% of patients 100 mg: 30% of patients	Placebo: 15% of patients 28 mg: 45% of patients 50 mg: 76% of patients
Fibrosis improvement without MASH worsening	Placebo: 22.4% of patients 25 mg: 9.6% of patients No effect if F4	No effect	Placebo: 29% of patients 120 mg: 48% of patients	Placebo: 14% of patients 80 mg: 24% of patients 100 mg: 26% of patients	Placebo: 20% of patients 28 mg: 37% of patients 50 mg: 41% of patients
References	(172)	(100, 101)	(173)	(174)	(175)

### 4.1 Obeticholic acid (OCA) and FXR agonists

OCA is a semi-synthetic 6α-ethyl derivative of the bile acid chenodeoxycholic acid, a first-in-class farsenoid-X receptor (FXR) agonist (159). FXR is a nuclear receptor highly expressed in the liver, but also in the small bowel, which is activated by bile acids. It has pleotrophic actions, modulating bile acids, cholesterol, and glucose metabolism, as well as lipogenesis (176). It also modulates vascular remodeling, inflammation (170, 171), fibrogenesis (177), and the intestinal barrier integrity (178, 179). FXR activation upregulates fibroblast growth factor-19 (FGF-19) and FGF-21, which also have direct anti-steatogenic and anti-fibrotic properties (180).

Preclinical studies in animal models of MASH suggested that OCA could be an effective treatment for improving steatohepatitis and fibrosis (165, 166, 181). After a proof-of-concept study in 64 T2DM patients with MASLD treated with OCA controlled with placebo showing a decrease in liver enzymes (182), a phase 2b trial, the FLINT trial, in 283 patients, brought huge enthusiasm towards OCA, because it was the first time a drug demonstrated an improvement in liver fibrosis in non-cirrhotic MASH (183). The study was even terminated earlier, with 64 patients not performing the programmed liver biopsy, because it reached the primary endpoint of improvement of at least 2 points in NAS score without worsening fibrosis. The study raised some concerns, however, since it induced pruritus in almost one-fourth of the patients, worsened insulin resistance and lipid profile with an increase in total and LDL-cholesterol, and a decrease in HDL-cholesterol (184). Having said that, the CONTROL study showed that the detrimental effect on the lipid profile could be prevented with concomitant treatment with atorvastatin (185). Importantly, studies in compensated cholestatic cirrhosis suggested that treatment with OCA increases almost 4-fold the risk of hepatic decompensation (186).

A phase 3 trial, with 2,477 patients enrolled, the REGENERATE, in pre-cirrhotic MASH, treated with OCA 10 or 25 mg, controlled with placebo, confirmed the positive results in 2 interim analyses at 18 months, the first analysis after histological evaluation by a single-pathologist (169) and the second one by a consensus panel of 3 pathologists (172). The analysis of 931 patients with fibrosis F2 or F3 showed a higher percentage of patients achieving fibrosis improvement (22% versus 10%), and MASH resolution (6.5% versus 3.5%) when treated with 25 mg of OCA compared to placebo (172). Liver stiffness and NITs for fibrosis decreased regardless of histologic response (187). Of note, pruritus occurred in one-third of the patients with the lowest dose and around half with the highest one. Dyslipidemia was very frequent in almost half the patients, and there was a slight increase in gallstone-related events. There were no differences between OCA and placebo in cardiovascular events (172).

A cost-effectiveness study suggested that treatment with OCA would only be cost-effective if the price of the drug would decrease by 78% (188).

A press release from September 2022 by Intercept Pharmaceuticals reported the results of the REVERSE study, a phase 3 study of OCA in compensated cirrhosis, which failed to improve fibrosis by histology, even though it had a positive impact on liver stiffness.

Two applications for FDA accelerated approval, after the first interim analysis in 2020 and, more recently, after the second in 2023, were denied on the basis of promising yet inconclusive benefits with safety concerns regarding pruritus, lipid profile, gallstone complications and rare drug-induced liver injury (DILI) in patients with advanced fibrosis or cirrhosis (189). In consequence, the company abandoned OCA as a treatment for MASLD.

Different non-bile acids FXR agonists either steroidal such as EDP-395 (190) and non-steroidal such as cilofexor (191) and tropifexor (192, 193) have been evaluated for MASH treatment, but results were not convincing, either alone or in combination with other drugs (194–196).

### 4.2 Lanifibranor and other PPAR agonists

PPARs are nuclear receptors that act as lipid sensors, being naturally activated by fatty acids or derivatives. PPARs are pleiotropic with multiple metabolic and immunomodulatory effects. There are different types of PPAR. PPAR-α is highly expressed in the liver, and its main action is the regulation of fatty acid oxidation. An example of PPAR-α agonists is fibrates, which are used to treat hypertriglyceridemia. PPAR-γ is highly expressed in the adipose tissue being crucial for adipocyte differentiation, and promotes whole-body glucose tolerance by promoting repression of adipocyte lipolysis by insulin. An example of PPAR-γ is thiazolidinedione used to treat T2DM. PPAR-δ is highly expressed in the liver and the muscle, where it modulates glucose metabolism towards a less glycolytic and more oxidative profile, improving lipid and glucose metabolism (197).

Lanifibranor is a pan-PPAR agonist that showed, in multiple preclinical models of MASH, to improve liver histology including fibrosis, as well as promote weight loss, and better glucose and lipid profile (198). A phase 2b study, the NATIVE, in 247 patients with non-cirrhotic MASH, showed promising results, with a higher percentage of patients with MASH resolution without worsening fibrosis (9% for placebo vs. 25% for 800 mg/day vs. 35% for 1,200 mg after 24 weeks) and with fibrosis improvement without worsening MASH (29% vs. 39% vs. 48%). It was well tolerated, with an increase in adiponectin despite weight gain, and an improvement in lipid profile (173). A phase 3 study (NCT04849728), NATIV3, is currently ongoing, which evaluates a 72-week treatment with lanifibranor 800 mg/day or 1,200 mg/day, controlled for placebo in non-cirrhotic patients with MASH and significant fibrosis (F2 or F3). It is expected to be finished in 2026.

Saroglitazar is a dual PPAR-α/γ agonist that promotes insulin resistance and protects from atherogenic dyslipidemia promoting a decrease in small dense LDL and triglycerides (158, 168, 199). Small phase 2 and observational studies suggest saroglitazar has beneficial effects in steatosis, MASH and fibrosis by NITs (167, 199–201), which places saroglitazar as a promising drug. Phase 2b studies with histological endpoints are ongoing (NCT02704403).

### 4.3 Resmetiron and other thyroid-mimetics

MASLD is associated with hypothyroidism (161, 162), and particularly relative intrahepatic hypothyroidism (202), through a shift in conversion of T4 to the inactive hormone rT3 as opposed to the active T3, as well as a decrease in the hepatic expression of THR (160, 202).

There are two THR: THR-β which mediates the beneficial effects in lipid metabolism, and THR-α which mediates the adverse effects, including cardiac effects. Resmetirom is a THR-β agonist with specific uptake into the liver (203). Preclinical studies in animal models of MASH showed histological benefits in the liver, and improvement of glucose and lipid metabolism, independent of body weight (204).

A phase 2b study (174) in 125 patients with MASH and LFC at least 10% by MRI-PDFF, treated with resmetirom 80 mg or placebo, during 36 weeks, showed a relative decrease of the LFC of 37% compared to 8% with placebo, which is higher than the anti-steatogenic effects of OCA or lanifibranor. A higher proportion of patients achieved MASH resolution (27% vs. 6%), but there was no difference in fibrosis improvement assessed by histology, even though there was an improvement in NIT such as ELF, Pro-C3, and transient elastography (205). Resmetirom was well tolerated and induced advantageous changes in lipid profile, decreasing LDL-cholesterol, triglycerides, and lipoprotein(a) (174).

Preliminary results of a phase 3 trial were recently presented at the EASL meeting, the MAESTRO-NASH, which evaluated the effect of 52 weeks of treatment with resmetirom 80 mg or 100 mg, compared with placebo, in 966 patients with fibrosing, non-cirrhotic MASH (F1-F3). There were dose–response increased proportions of patients achieving MASH resolution without worsening of fibrosis (10% vs. 26% vs. 30%) and of fibrosis improvement without worsening of NAS score (14% vs. 24% vs. 26%). It was also associated with impressive improvements in steatosis, NIT assessed fibrosis, liver and spleen volume (206).

Three other phase 3 studies are currently ongoing: MAESTRO-NAFLD-1 and its open-label extension MAESTRO-NAFLD-OLE, and MAESTRO-NASH-outcomes in cirrhotic patients.

VK2809 is another THRβ liver-specific agonist, which showed in phase 2a and 2b in fibrotic MASH, the VOYAGE study, reductions in LFC after 12 weeks. We are still waiting for the histologic results after 52 weeks.

### 4.4 Efruxifermin and other FGF-21 agonists

FGF-21 orchestrates energy metabolism, promoting fatty acid oxidation in the liver, insulin sensitivity in peripheral tissues, and acting in the brain decreasing sweet and alcohol preference. Furthermore, even though it is a non-mitogenic hormone, it promotes tissue repair, blunting oxidative stress, inflammation, and fibrogenesis (207).

Efruxifermin is a fusion protein of the human IgG1 Fc domain linked to modified human FGF-21, with a long half-life allowing weekly administration. A phase 2a trial, the BALANCED trial, in 80 patients with non-cirrhotic, fibrotic (F1-F3) MASH, evaluated the effect of 16 weeks of treatment with ascending doses of efruxifermin, controlled with placebo (208). Treatment resulted in a dose–response decrease in relative LFC, over 70% for the highest doses. It also resulted in a decrease in NIT of liver fibrosis ProC3 and ELF score (208). A second phase 2a study in 30 patients with compensated MASH-associated cirrhosis also showed it to be safe, and to result in improvement of glucose metabolism (lowering glycated hemoglobin) and NIT of fibrosis (ELF, FAST score, and ProC3) (209). Of note, treatment with efruxifermin was associated with the development of anti-drug antibodies in 72% of the patients, though its clinical implications are yet to be known (208).

A phase 2b study, the HARMONY, enrolled 128 patients with non-cirrhotic, fibrotic (F1-F3) MASH patients treated with 28 mg or 50 mg of efruxifermin per week or placebo, for 96 weeks. Treatment resulted in a dose–response increase in the proportion of patients achieving fibrosis improvement without worsening of MASH (39 and 41% vs. 20% for placebo), and MASH resolution without worsening of fibrosis (47 and 76% vs. 15% for placebo) (175).

A phase 3 program consisting of 3 trials, SYNCHRONY Histology, SYNCRONY Real-World, and SYNCHRONY Outcomes is currently undergoing to evaluate safety and efficacy of efruxifermin in patients with MASH, including long-term clinical outcomes, according to Akero Therapeutics press release, November 13, 2023.

Two other FGF-21 agonists are under investigation: pegbelfermin and pegozafermin. Both are pegylated FGF-21 agonists. Regarding pegbelfermin, after a small phase 2a study in patients with T2DM and obesity showing a relative decrease of MRI-PDFF up to 56% after 12 weeks of treatment (156), two phase 2b studies, the FALCON-1 and -2 in patients with MASH and advanced fibrosis (F3) or compensated cirrhosis, respectively, confirmed efficacy in decreasing LFC and NIT of fibrosis but did not achieve histological fibrosis improvement (163, 210). Pegozafermin was first evaluated in a 12-week phase 1/2 study in patients with phenotypic MASH diagnosed by transient elastography of ≥7 kPa associated with central obesity and either T2DM or elevated aminotransferases, which resulted in decreased liver enzymes and MRI-PDFF (164). A phase 3b study, the ENLIVEN trial, in 219 patients with MASH and significant fibrosis (F2-F3), compared placebo with different doses of pegozafermin (211). Pegozafermin treatment resulted in a higher proportion of patients achieving the primary endpoints fibrosis improvement without worsening MASH and MASH resolution without fibrosis worsening. It is also associated with a decrease of MRI-PDFF up to 50% (lower than the one achieved with efruxifermin) and improvement in lipid profile (157).

### 4.5 Other strategies in development

FGF-19 is a gut hormone induced by FXR activation, which has beneficial effects in bile acids, carbohydrates, and energy homeostasis, being downregulated in patients with MASH (212). It has, however, a potential carcinogenic effect (213), promoting hepatocellular carcinoma and associating with worse prognosis (214). Adalfermin is an engineered FGF-19 analog that lacks tumorigenic potential due to a 5 amino-acids deletion in the amino-terminus (215) that results in the inability to activate STAT-3 (216). It showed beneficial histological effects in animal models of MASH (217). Phase 2a studies suggested that adelfermin could improve LFC and fibrosis by NITs and histology (218–220). A phase 2b study, ALPINE 2/3, in 171 patients with fibrotic (F1-F3) MASH, treated with increasing doses of adelfermin for 24 weeks, controlled for placebo, did not achieve its primary endpoint of fibrosis improvement without worsening of MASH, even though it did show a dose–response for resolution of MASH without worsening of fibrosis (221).

Strategies that act directly on lipid metabolism are ongoing. Different enzymes in lipogenesis have been targeted: acetyl-CoA carboxylase inhibitor firsocostat, fatty acid synthase inhibitor denifanstat, DGAT2 inhibitors, and stearoyl CoA-desaturase-1 inhibitor aramchol. All of them consistently showed improvements in LFC, in small trials (195, 222–225). Aramchol was evaluated in a phase 2b study, ARREST, in patients with MASH, which failed to achieve histological benefit in MASH or fibrosis (226). A phase 3 trial, ARMOR study (NCT04104321), is currently ongoing.

Anti-fibrotic strategies are still ongoing, even after the major failure of simtuzumab, a monoclonal antibody that binds to LOXL2 (an enzyme responsible for elastin and collagen crosslinking), and acts as an immunomodulator (155). Belapectin, a galectin-3 inhibitor, was evaluated in a phase 2b study in patients with MASH-associated cirrhosis with portal hypertension and found that in patients with esophageal varices, 52 weeks of treatment associated with a decrease in portal pressure and a decreased risk of new varices development (227). A phase 2/3 trial, the NAVIGATE (NCT0436868), for the prevention of esophageal varices in MASH-cirrhosis is ongoing.

Lastly, the field is also moving toward directed therapies with oligonucleotide-based therapies that target genes with variants initially identified in genome-wide association studies, to be associated with risk for MASH. For example, patients with PNPLA3 148 M variant, which has detrimental effects on lipid remodeling in hepatocytes, can be targeted for PNPLA3 therapeutic oligonucleotide inhibition, which is already ongoing. A similar approach could be offered for patients who do not present the protective loss-of-function variant on HSD17B13 (i.e., insertion of adenine in a donor splice site in exon 6) (228).

## 5 Conclusion

MASLD, the liver manifestation of adiposopathy, is a condition that is associated with increased all-cause mortality, but only a minority of patients will progress to end-stage liver disease. As such, adiposopathy and lifestyle exercise-promoting interventions should be offered to all patients.

Weight loss is the most efficient strategy, and all tools must be considered, such as lifestyle intervention, bariatric surgery and endoscopy, and most recently, the growing panoply of highly efficient anti-obesity drugs.

Intense research is being performed to find a drug that acts specifically in liver disease, for those patients with fibrotic MASH that are at-risk of progressive liver disease. The pathway for MASH-effective drug discovery is not easy, but 3 strong candidates are already in advanced research, namely resmetirom, lanifibranor, and efruximin.

The near future holds for a shift in the paradigm of MASH treatment, possibly with combination and precision therapy targeting particular deranged pathways for each patient.

## Author contributions

MVM: Conceptualization, Writing – original draft, Writing – review & editing.

## Glossary

ASK-1: Apoptosis signal-regulating kinase-1
BMI: Body mass index
CCR: Chemokine receptor
FGF: Fibroblast growth factor
FXR: Farsenoid-X receptor
GIP: Glucose-dependent insulinotropic polypeptide
GLP-1: Glucacon-like peptide-1
LFC: Liver fat content
LOXL-2: Lysyl oxidase-like-2
MASH: Metabolic associated steatohepatitis
MASH: Metabolic-associated steatohepatitis
MASLD: Metabolic-associated steatotic liver disease
MD: Mediterranean diet
NIT: Non-invasive tests
NNT: Number needed to treat
OCA: Obeticholic acid
PPAR: Peroxisome proliferated-activated receptor
ProC3: Propeptide of type III collagen
STAT-3: Signal transducer and activator of transcription-3
T2DM: Type 2 diabetes mellitus
